# Icariin improves cardiac function and remodeling via the TGF-β1/Smad signaling pathway in rats following myocardial infarction

**DOI:** 10.1186/s40001-023-01588-4

**Published:** 2023-12-19

**Authors:** Ji Jia, Xing-an Zhao, Si-ming Tao, Jun-wen Wang, Rong-liang Zhang, Hua-lei Dai, Xin-jin Zhang, Ming-hua Han, Bei Yang, Yu Li, Jin-tao Li

**Affiliations:** 1 Department of Cardiology, Affiliated Hospital of Yunnan University, Kunming, 650021 Yunnan China; 2 Dali University, Dali, 671003 China; 3 West China Hospital, Sichuan University, Chengdu, 610041 China; 4 Department of Echocardiography, Affiliated Hospital of Yunnan University, Kunming, 650021 China; 5 Kunming Medical University, Kunming, 650500 China

**Keywords:** Icariin, Myocardial infarction, Ventricular remodeling, Myocardial fibrosis, TGF-β1/Smad signaling pathway

## Abstract

**Background:**

Postinfarction cardiac remodeling presents a compensatory mechanism aimed at mitigating congestive heart failure. It is distinguished by progressive dilatation and hypertrophy of the ventricular chambers, fibrotic alterations, and prolonged apoptosis of cardiomyocytes. The primary objective of this study was to assess the effects of icariin on myocardial fibrosis and ventricular remodeling in rats subjected to myocardial infarction (MI).

**Methods:**

Male Sprague‒Dawley (SD) rats were subjected to randomization and subsequently divided into distinct groups: the control group, the sham group (undergoing sham operation), the MI group (experiencing ligation of the left anterior descending artery), and the icariin group. Within the icariin group, rats were further categorized into three different dose groups based on the administered icariin dosage: the MI30 group (30 mg/kg/day), the MI60 group (60 mg/kg/day), and the MI120 group (120 mg/kg/day). Cardiac function evaluation was carried out using echocardiography. Histological examinations, including hematoxylin and eosin (HE) staining, Masson staining, and immunohistochemistry studies, were conducted 90 days after the occurrence of MI. Additionally, Western blotting was employed to assess TGF‐β1, p-Smad2, and p-Smad3 levels.

**Results:**

The administration of icariin revealed a noteworthy enhancement in cardiac function among rats afflicted with left anterior descending coronary artery (LAD) ligation. In comparison to the icariin groups, the MI group exhibited reduced EF and FS, along with elevated LVEDD and LVESD. Furthermore, the cardiac fibrosis levels in the MI group rats exhibited a considerable increase compared to those in the icariin group. Notably, the levels of Collagen I, Collagen III, MMP2, and MMP9 were significantly higher in the MI group than in the icariin group, with evident distinctions. Moreover, the expression levels of TGF-β, IL-13, p-Smad2, and p-Smad3 were notably upregulated in the MI group compared to the icariin group.

**Conclusions:**

In an experimental rat model of MI, the administration of icariin resulted in the amelioration of both cardiac function and remodeling processes, operating through the intricate TGF-β1/Smad signaling pathway.

## Introduction

As the standard of living steadily improves, the aging population is on the rise, and myocardial infarction (MI) stands as the foremost contributor to both morbidity and mortality within the realm of cardiovascular diseases worldwide [1]. While modern reperfusion therapy serves as the most efficacious approach for diminishing infarct size and ameliorating clinical outcomes after MI, heart failure remains the primary cause of death subsequent to MI. The process of heart remodeling following MI is acknowledged as the initial phase leading toward heart failure [2, 3]. Consequently, the reversal of ventricular remodeling emerges as a highly desirable prospect for the treatment of MI. Certain angiotensin-converting enzyme inhibitors (ACEIs) and angiotensin receptor blockers (ARBs), when administered as long-term treatment, have demonstrated the capacity to attenuate ventricular remodeling [4–6]. However, a significant number of patients encounter intolerance to ACEIs/ARBs due to conditions such as renal dysfunction, hypotension, or hyperkalemia, among others. Hence, there exists considerable interest in identifying novel therapeutic agents capable of enhancing ventricular remodeling, thereby addressing this pressing concern.

Myocardial fibrosis and ventricular remodeling have been identified as crucial pathological factors contributing to unfavorable outcomes following MI [7]. Of notable significance, the TGF‐β1/Smad signaling pathway exhibits a close association with both myocardial fibrosis and ventricular remodeling [8, 9]. Multiple studies have revealed that TGF-β1/Smad signaling plays a pivotal role in promoting renin–angiotensin–aldosterone system (RAAS) activation and modulating the expression of matrix metalloproteinases (MMPs) [10, 11]. Through binding to the TGF-β1 receptor on the plasma membrane, it facilitates the phosphorylation of Smad2/3, leading to their interaction with Smad4 to form a complex that translocates into the nucleus, thereby initiating gene transcription [12]. Numerous experimental investigations have demonstrated a marked overexpression of TGF-β1 in the myocardium after MI, and a noteworthy reduction in MI-induced fibrosis within the heart has been observed upon inhibition of its signaling pathway [13]. Consequently, targeting the TGF‐β1/Smad signaling pathway holds promising potential as a therapeutic strategy for addressing myocardial fibrosis and ventricular remodeling.

China has used traditional Chinese medicine (TCM) for more than 2000 years with proven curative effects, and the rapid development of the Chinese medicine industry has been attributed to China's high regard for Chinese medicines [14, 15]. Icariin is part of the Epimedium Genus of TCM [16]. Scientific investigations have highlighted a multitude of properties associated with components of Epimedium, and icariin, in particular, has exhibited anti-inflammatory, antioxidant, immunomodulating, and antitumor activities [17, 18]. Notably, in animal studies, icariin has demonstrated neuroprotective and cardioprotective effects [19, 20]. The current study delves into the exploration of icariin's potential mechanism in mitigating ventricular remodeling within an experimental rat model of MI, in pursuit of new potential therapeutic targets to prevent the progression of myocardial infarction patients towards myocardial fibrosis, ventricular remodeling, and ultimately heart failure.

## Materials and methods

### Animals and surgery

Adult male Sprague–Dawley (SD) rats weighing 220–300 g were procured from the Experimental Animal Center of Yunnan University College of Medicine. Prior to commencement, the study received ethical approval from the Animal Research Committee of Yunnan University College of Medicine under approval number YNU20220333. The rats were housed in a controlled environment maintained at 20 ± 2 °C with a 12-h light/dark cycle and 60% humidity. Following a 1-week acclimation period, the rats were randomly allocated to four groups: the control group, the sham group, the MI group, and the icariin group. In the icariin group, rats were administered icariin at doses of 30, 60, and 120 mg/kg/day for 14 consecutive days. Consequently, within the icariin group, the rats were further subdivided into three subgroups named MI 30, MI 60, and MI 120 based on the dosage they received. Anesthesia was induced using 1% pentobarbital sodium at a dose of 50 mg/kg through intraperitoneal injection, after which the rats were connected to an electrocardiograph. Subsequently, the rats’ neck skin was incised, and a step-by-step approach was employed to perform thoracotomy, separating the fascia layer by layer. The rats were then intubated and connected to a ventilator. MI was induced by ligating the left anterior descending coronary artery (LAD) following heart exposure. The same surgical procedure was performed in the sham operation group, with the exception of LAD coronary artery ligation. The successful induction of myocardial ischemia was further indicated by the presence of ST-segment elevation in the electrocardiography records.

### Histopathological and immunohistochemical staining

Ninety days postsurgery, the rats were euthanized, and relevant samples were collected. During blood collection, heart tissues were also procured for histopathologic examination. Subsequently, the heart tissues were subjected to formalin fixation, paraffin embedding, and sectioning at a thickness of 4 μm. Hematoxylin–eosin (H&E) staining was performed on the tissue sections. TGF-β and IL-13 expression in the serum was assessed using the enzyme-linked immunosorbent assay (ELISA) method.

### Masson’s trichrome staining

Collagen deposition and fibrosis were evaluated through Masson trichrome staining, employing the standard methodology. Left ventricular tissues procured from euthanized rats were subjected to fixation in 4% paraformaldehyde at 4 °C for 48 h, followed by paraffin embedding and sectioning at a thickness of 4 µm. The Masson staining assay was conducted utilizing a Masson Stain Kit (Sigma-Aldrich, Cat. No. HT15-1KT) in accordance with the provided instructions.

### Immunohistochemical staining

Heart tissue sections measuring 4 μm in thickness were meticulously prepared for immunohistochemical staining, following established procedures. Subsequent to deparaffinization and tissue rehydration, a 3% hydrogen peroxide/methanol solution (provided by Tianjin Wind Boat Chemical Reagent Technology Co., Ltd) was employed to quench endogenous peroxidase activity. The sections were further subjected to incubation in a 10 mM citrate buffer solution (pH 6.0, sourced from Thermo Fisher Scientific) and subjected to antigen retrieval via autoclave sterilization. Following cooling and blocking, primary antibodies targeting collagen I, collagen III, MMP2, and MMP9 (sourced from Abcam, Cambridge, MA, USA) were applied. Subsequently, tissue sections were visualized using the 3,3′-diaminobenzidine tetrahydrochloride detection kit (BioGenex, CA, USA) and counter-stained with hematoxylin (Sigma-Aldrich).

### Cell culture

The murine cell line RAW264.7, obtained from CHI Scientific Inc. (Jiangsu, China, Cat. No. 7-1115), were cultured routinely in Dulbecco's Modified Eagle's Medium (DMEM, Gibco, Cat. No. 11965-092) supplemented with 10% fetal bovine serum (FBS, Sigma-Aldrich, Cat. No. F2442) and 1% penicillin–streptomycin (Gibco, Cat. No. 15140-122) at a temperature of 37 °C with 5% CO_2_. For cell dissociation, 0.25% trypsin and 0.02% EDTA were utilized. In the experimental procedures, cells from passages 3 to 6 were employed. Under interleukin-4 (IL-4) stimulation, RAW264.7 cells were differentiated into M2-type macrophages (M2). Cryopreservation of these cells was carried out using a solution containing 10% dimethyl sulfoxide (DMSO). The cultured RAW264.7 cells were randomly divided into distinct groups: (1) RAW264.7; (2) RAW264.7 + IL-4; (3) RAW264.7 + IL-4 + DMSO; and (4) RAW264.7 + IL-4 + icariin. The RAW264.7 group functioned as the untreated control, devoid of any pharmacological intervention. Conversely, the remaining experimental groups were subjected to distinct treatments, including the administration of 20 ng/mL recombinant IL-4 (SinoBiological, Cat. No. 51084-M08B), IL-4 in conjunction with DMSO, and IL-4 co-administered with 60 μM icariin (Aladdin, Shanghai, China, Cat. No. I141014-1 g).

### Western blotting analysis

Proteins were extracted from cryopreserved cardiac tissues maintained under liquid nitrogen storage conditions, utilizing a RIPA lysis buffer. Quantification of protein concentration was carried out employing the BCA protein assay reagent kit, sourced from the Beyotime Institute of Biotechnology, Shanghai, China. In this analysis, the primary antibody concentrations employed were as follows: TGF‐β1 (1:1000, Abcam, ab215715), p-Smad2 (1:1000, Abcam, ab280888), p-Smad3 (1:2000, ab52903), and β-actin (1:1000, Abcam, ab8226). Detection of the protein bands was facilitated through the utilization of an enhanced chemiluminescence (ECL) system, with subsequent quantification being executed utilizing Image J software.

### Echocardiographic studies

Cardiac echocardiography was conducted at multiple time points for the experimental rats, including baseline (before the surgical procedure), as well as 15, 30, 60, and 90 days after surgery. Before the examination, the rats were anesthetized using 3% pentobarbital sodium. The chest of each animal was shaved, and the animals were positioned in a dorsal decubitus posture. Commercially available instruments (Vivid T8, GE Healthcare, United States), M-mode, two-dimensional, and Doppler echocardiography were performed. During the echocardiographic assessments, we determined several key parameters, including the left ventricular end-diastolic diameter (LVEDD, mm), left ventricular end-systolic diameter (LVESD, mm), left ventricular shortening fraction (FS, %), and left ventricular ejection fraction (EF, %).

### Statistical analysis

The data are presented as the means ± standard deviation (SD). Comparisons among multiple groups were conducted using one-way analysis of variance (ANOVA), followed by post hoc comparisons employing Tukey's multiple comparison test. GraphPad Prism 8.0 (GraphPad Software, San Diego, CA, USA) was utilized for all statistical analyses. A significance level of *P* < 0.05 was considered indicative of statistical significance between the groups.

## Results

### Icariin treatment improves left ventricular remodeling in rats with MI

**Fig. 1:**
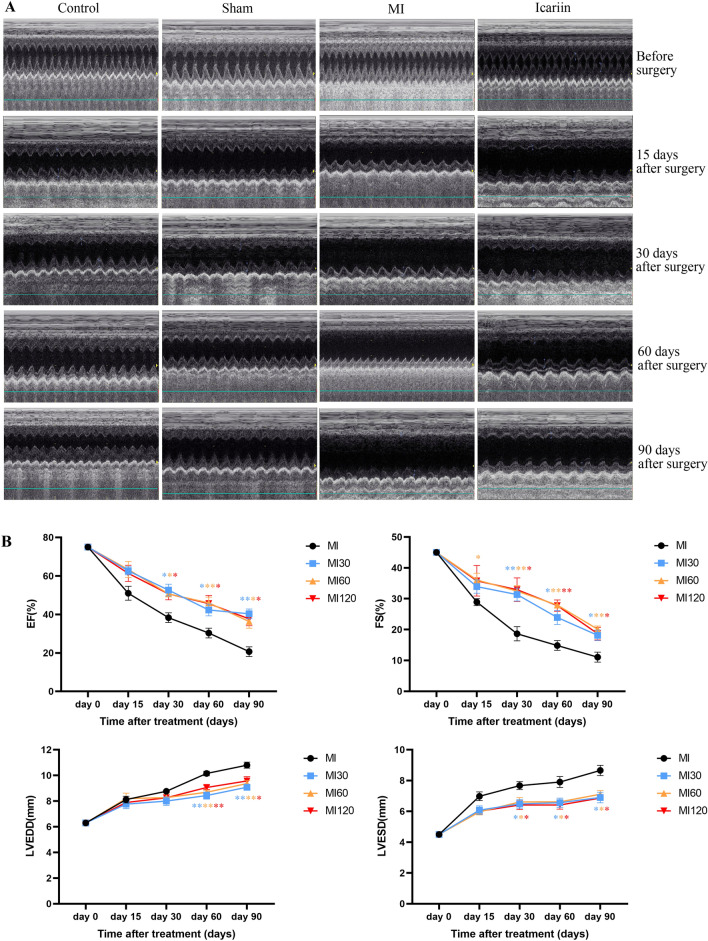
The echocardiogram results showed a significant improvement in cardiac function in rats after icariin treatment. **A** M-mode echocardiography was used to capture representative images of SD rats before and after myocardial infarction (MI) in the icariin treatment group, MI group, sham group, and control group. **B** The left ventricular ejection fraction (EF), left ventricular fractional shortening (FS), left ventricular end-diastolic diameter (LVEDD), and left ventricular end-systolic diameter (LVESD) were subjected to comparative analysis among different MI groups receiving various dosages of icariin at 0, 15, 30, 60, and 90 days after MI. *P < 0.05, **P < 0.01

Cardiac function assessment was carried out at multiple time points, including baseline and 15, 30, 60, and 90 days post-myocardial infarction (MI) surgery. Echocardiograms were utilized to quantify parameters such as LVESD, LVEDD, EF, and FS in rats. The M-mode graphs of each experimental group are presented in Fig. 1A. Comparing the MI and icariin treatment groups to the control and sham groups, a significant reduction in EF and FS, along with notable increases in LVEDD and LVESD, were observed. Nevertheless, treatment with icariin facilitated the functional recovery of postischemic hearts, as evidenced by increased EF and FS, coupled with decreased LVEDD and LVESD, relative to the MI group. Furthermore, an exploration of the impact of icariin concentration on left ventricular remodeling was undertaken. Post hoc multiple comparisons further revealed significant distinctions between the icariin treatment groups and the MI group. On the 15th day post-operation, there were no statistically significant differences in EF values among the four groups. However, on the 30th day post-operation, when compared to the MI group, the MI30, MI60, and MI120 groups receiving icariin treatment exhibited significantly elevated EF values, with respective P-values of 0.0125, 0.0275, and 0.0211. The EF values for the MI30, MI60, and MI120 groups exhibited statistically significant increases relative to the MI group on the 60th day (*P* < 0.05, *P* < 0.01, *P* < 0.05, respectively) and the 90th day (*P* < 0.01, *P* < 0.05, *P* < 0.05, respectively). Regarding the FS (Fractional Shortening) values, it is observed that the MI30, MI60, and MI120 groups all demonstrated significantly higher FS values compared to the MI group. When comparing the values on the 30th postoperative day, the respective *P* values were (*P* < 0.01, *P* < 0.01, *P* < 0.05). On the 60th day post-operation, the respective *P*-values were (*P* < 0.05, *P* < 0.01, *P* < 0.01), and on the 90th day post-operation, the respective *P* values were (*P* < 0.05, *P* < 0.01, *P* < 0.05). Similarly, at 60 and 90 days postoperatively, LVEDD and LVESD were notably higher in the icariin group compared to the MI group (*P* < 0.05). However, no statistically significant differences in EF, FS, LVEDD, and LVESD were observed among the three different icariin dosage groups. In conclusion, the present study demonstrated the development of cardiac dysfunction in MI rats, while icariin administration effectively ameliorated heart function.

### Icariin reversed aggravation of myocardial ischemia

**Fig. 2:**
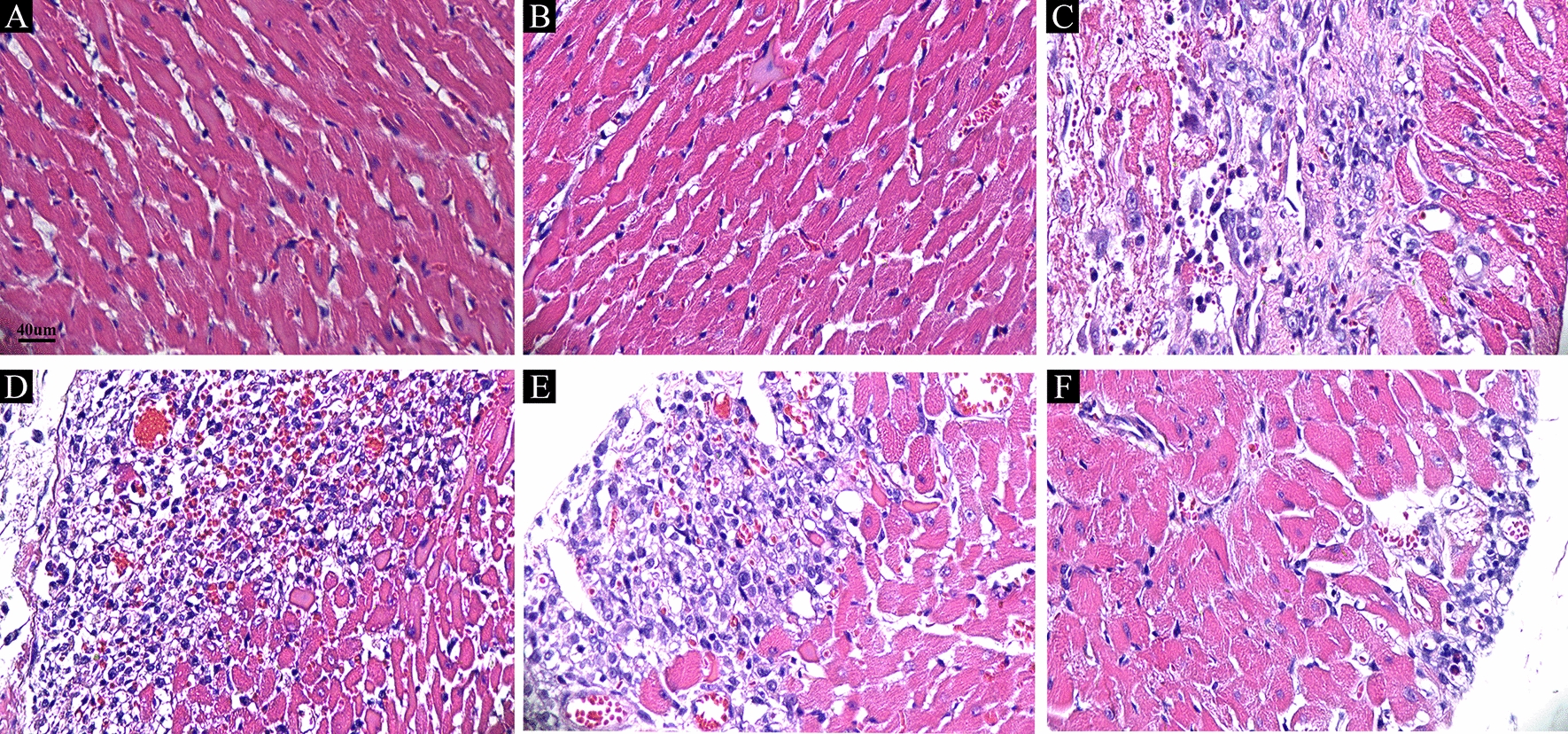
Oral administration of icariin attenuated myocardial ischemic injury and diminished the histological lesions. Representative photos of heart sections stained with H&E. **A** The control group, **B** The sham group, **C** The MI group, **D** The MI 30 group, **E** The MI 60 group, **F** The MI 120 group. (magnification, × 40; scale bar, 250 μm, with black arrows pointing to infiltrating immune cells)

In this study, we examined postischemic MI and cardiac structural alterations using hematoxylin and eosin staining at the 90-day mark following coronary ligation. In the Control and Sham groups, the myocardial tissue structure in rats exhibited a dense and orderly pattern. Myocardial fibers appeared intact, without any signs of myocardial fiber rupture or infiltration of inflammatory cells (Fig. 2A, B). In the MI, MI30, MI60, and MI120 groups, disruptions in myocardial fiber integrity and disorganized arrangement of fibers were evident. Some myocardial cells showed nuclear dissolution and fragmentation, accompanied by a substantial infiltration of inflammatory cells surrounding necrotic myocardial cells. In contrast, the icariin treatment group exhibited a reduction in infarct size compared to the MI group. Additionally, it maintained a better structural integrity of myocardial fibers and reduced the infiltration of inflammatory cells (Fig. 2C-F). These results indicate that icariin effectively ameliorated the pathological changes associated with MI.

### Icariin improved myocardial fibrosis

**Fig. 3:**
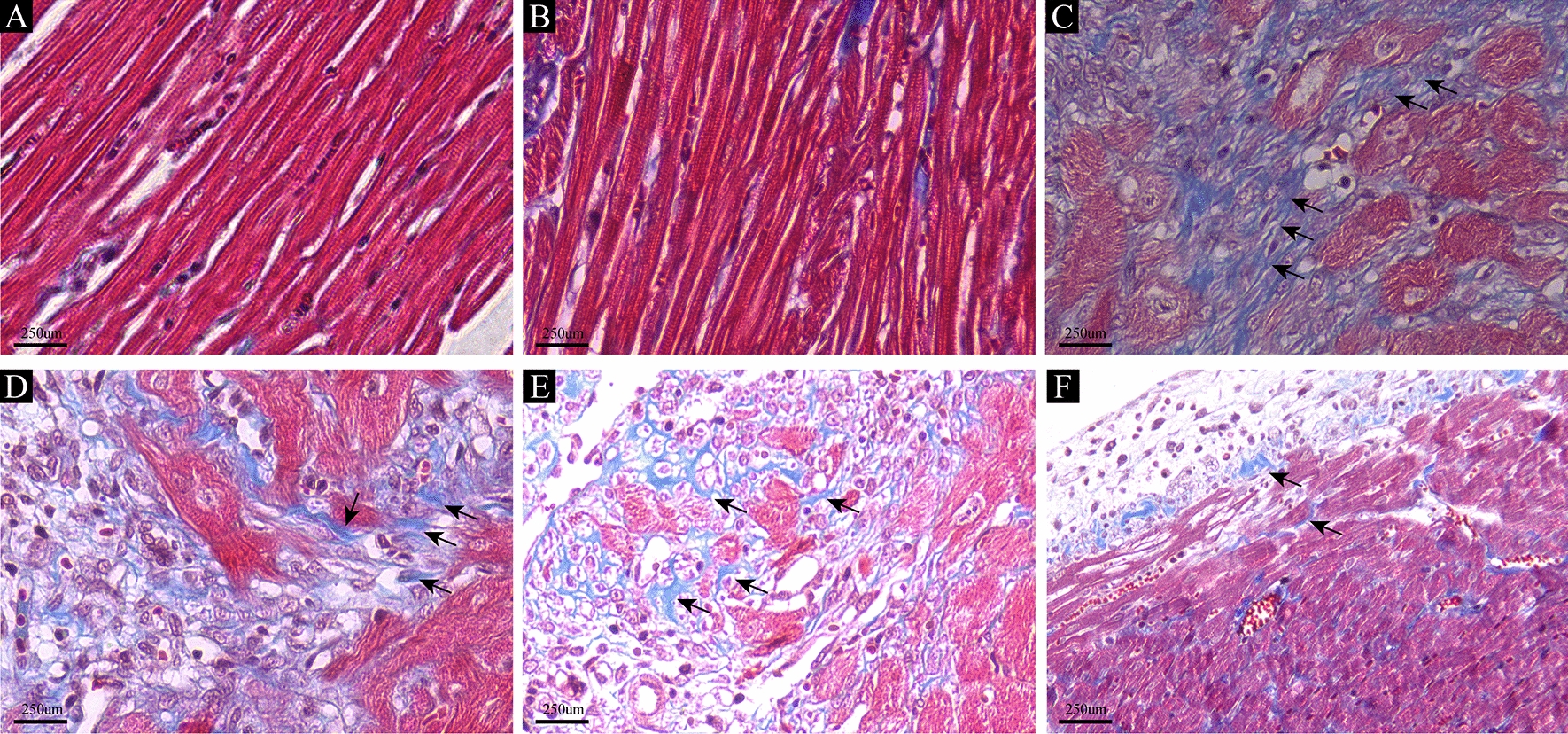
Icariin inhibited myocardial fibrosis. Masson trichrome staining revealed that the icariin group had less fibrous connective tissue than the MI group. **A** The control group, **B** The sham group, **C** The MI group, **D** The MI 30 group, **E** The MI 60 group, **F** The MI 120 group. (magnification, × 40; scale bar, 250 μm. The regions indicated by the black arrows correspond to collagen stained in blue)

Masson's trichrome staining was utilized to visualize collagen, which is indicated by blue staining. As depicted in Fig. 3, rats with myocardial infarction exhibited notably severe myocardial fibrosis, extensive collagen deposition areas, and significant cardiac disarray remnants. In contrast, both the control and sham groups exhibited distinct myocardial tissue structures with well-arranged myocardial cells. Notably, the icariin-treated group displayed significantly less myocardial fibrosis than the MI group.

### Icariin regulated the expression of TGF-β and IL-13 in ischemic tissue

**Fig. 4:**
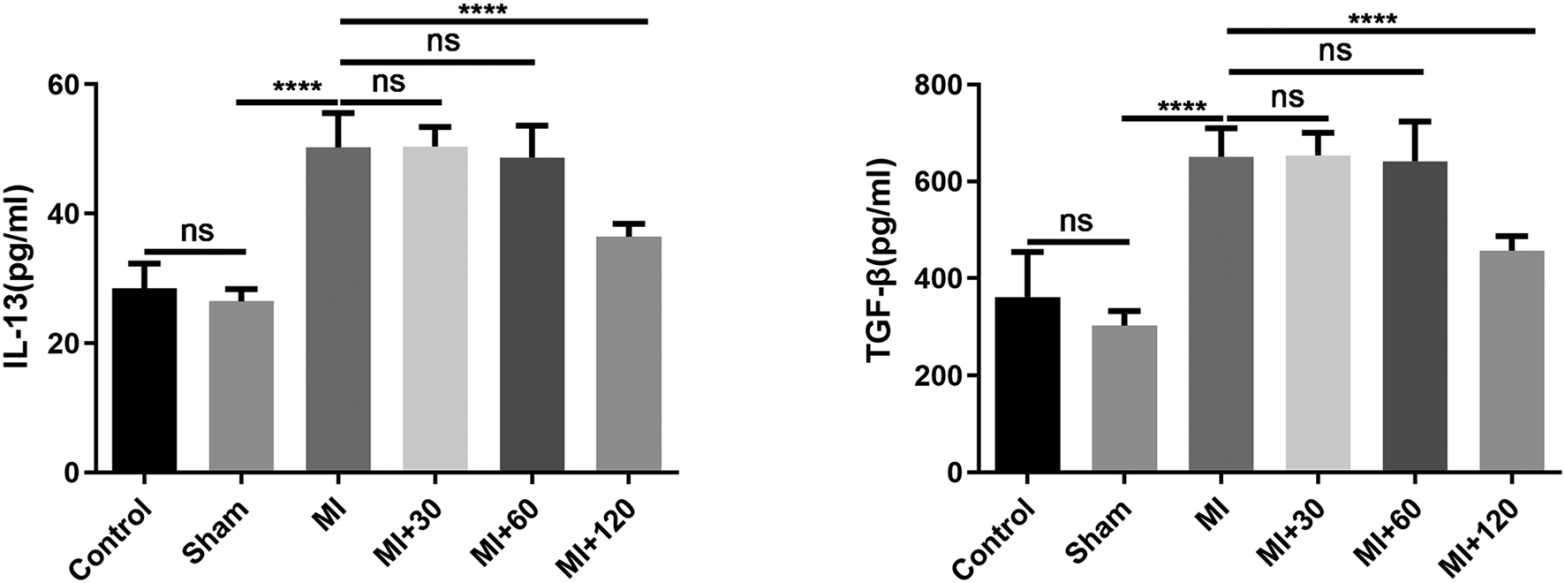
The bar graph depicts the quantitative analysis of TGF-β and IL-13, presented as the means ± SDs. Statistical analysis was performed using one-way analysis of variance (ANOVA) followed by Tukey's multiple comparison test, ****P < 0.0001

Cardiac ischemia is among the most prevalent factors contributing to myocardial fibrosis. The cytokine interleukin-13 (IL-13) plays a pivotal role in inflammation and fibrosis, as well as fostering the proliferation of T-lymphocytes. In the context of fibrogenesis, transforming growth factor beta (TGF-β) operates as a crucial molecular mediator through its intracellular signaling pathways. In this study, the expression levels of TGF-β and IL-13 were utilized as indicators to assess myocyte apoptosis following MI. Additionally, blood specimens were collected at the 90-day mark postsurgery, and the serum concentrations of TGF-β and IL-13 were assessed using enzyme-linked immunosorbent assay (ELISA). The IL-13 levels (pg/ml) in the Control and Sham groups were 28.40 ± 3.89 and 26.48 ± 1.86, respectively. In the MI, MI30, MI60, and MI120 groups, the IL-13 levels were 50.22 ± 5.36, 50.40 ± 3.01, 48.67 ± 4.97, and 36.42 ± 1.99, respectively. IL-13 was significantly elevated in the MI, MI30, MI60, and MI120 groups compared to the Control and Sham groups (P < 0.0001). However, there were no statistically significant differences in IL-13 levels among the MI, MI30, and MI60 groups (see Fig. 4). In contrast, the MI120 group exhibited a significant reduction in IL-13 levels compared to the MI, MI30, and MI60 groups (P < 0.0001). Similar to the results for IL-13, the statistical analysis for TGF-β (pg/ml) revealed the following values: in the Control and Sham groups, TGF-β levels were 360.7 ± 93.55 and 303.2 ± 29.96, respectively. In the MI, MI30, MI60, and MI120 groups, the TGF-β levels were 651.3 ± 58.52, 653.4 ± 46.74, 641.8 ± 81.97, and 457.1 ± 29.62, respectively. TGF-β was significantly elevated in the MI, MI30, MI60, and MI120 groups compared to the Control and Sham groups (P < 0.0001). However, there were no statistically significant differences in TGF-β levels among the MI, MI30, and MI60 groups. In contrast, the MI120 group exhibited a significant reduction in TGF-β levels compared to the MI, MI30, and MI60 groups (*P* < 0.0001). These findings collectively indicate that icariin effectively inhibits myocardial fibrosis while concurrently reducing the levels of TGF-β and IL-13.

### Icariin reduced the levels of collagen I, collagen III, MMP2, and MMP9

**Fig. 5:**
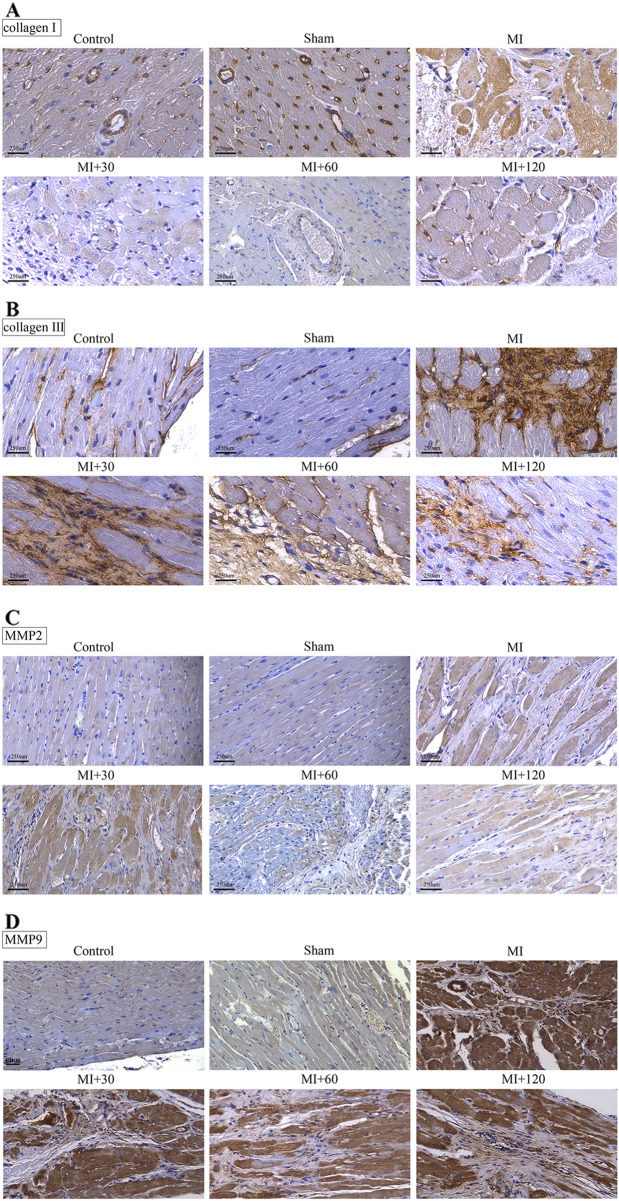
Icariin reduced the levels of collagen I, collagen III, MMP2 and MMP9. Immunohistochemical staining of collagen I (**A**), collagen III (**B**), MMP2 (**C**) and MMP9 (**D**) in heart sections. (magnification, × 40; scale bar, 250 μm)

Collagen I and collagen III represent predominant components of the intracardiac collagen matrix. MI induced a substantial increase in the deposition of collagen types I and III, as observed in both the MI and icariin treatment groups compared to the sham group. Conversely, the sham and control groups displayed minimal evidence of collagen I and collagen III deposition. Notably, the administration of icariin effectively attenuated the levels of collagen I and collagen III (see Fig. 5A, B). Furthermore, the study also investigated the expression of matrix metalloproteinase 2 (MMP2) and matrix metalloproteinase 9 (MMP9) proteins in various groups. MMPs are zinc-dependent endopeptidases that play a pivotal role in degrading collagen and aggrecan within the extracellular matrix (ECM). Among them, MMP2 and MMP9 are members of the matrix metalloproteinase family and are recognized for their critical involvement in processes such as cell invasion, metastasis, and angiogenesis. Angiogenesis, known as a key regenerative event, holds the potential to restore blood supply and repair cardiac function in patients with acute myocardial infarction (AMI). Immunohistochemical staining was employed to assess the expression of MMP2 and MMP9 in the ischemic myocardium. The findings unveiled increased levels of MMP2 and MMP9 in the MI group compared to the sham group. Conversely, the icariin treatment group exhibited a significant decrease in MMP2 and MMP9 levels relative to the MI group (see Fig. 5C, D). These outcomes suggest that icariin administration promotes blood perfusion recovery and fosters the formation of blood vessels in ischemic rats.

### Icariin inhibited the fibrosis of cultured cardiac cells by suppressing the TGF-β1/Smad signaling pathway

**Fig. 6:**
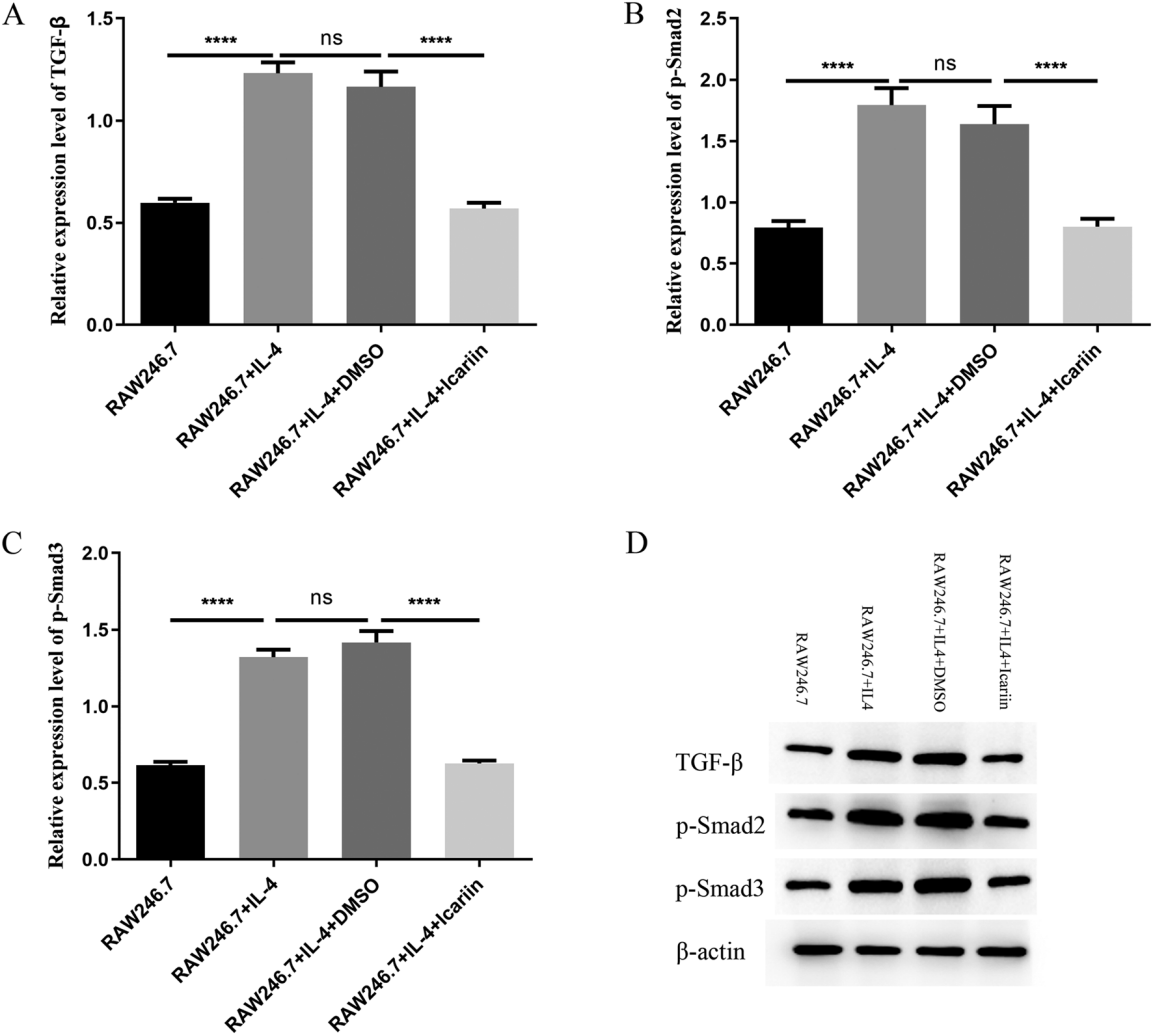
Western blot analysis of TGF‐β, p-Smad2, and p-Smad3. The bar graph shows quantitative analysis of the TGF‐β, p-Smad2, p-Smad3 and β-actin proteins, and the results were normalized to the RAW246.7 group (**A**–**C**). TGF‐β, p-Smad2, p-Smad3 and β-actin protein expression in different groups was detected by Western blot (**D**). The data are shown as the means ± SDs, one-way ANOVA followed by Tukey's multiple comparison test, ****P < 0.0001

As previously elucidated, the TGF-β1/Smad signaling pathway assumes a critical role in the pathogenesis of cardiac fibrosis. Notably, Smad2 and Smad3 serve as essential receptors during the initial stages of this signaling cascade. Upon activation by TGF‐β1, phosphorylated Smad2 (p‐Smad2) and phosphorylated Smad3 (p‐Smad3) play a key role in inducing cardiac fibrosis. To further substantiate the impact of icariin on cardiac fibrosis, RAW264.7 cells were exposed to IL-4 to induce the polarization of M2-type macrophages. In this context, the protein expression levels of TGF‐β1, p-Smad2, and p-Smad3 were assessed using Western blotting techniques. The results from Western blot analysis demonstrated a substantial increase in TGF‐β1, p-Smad2, and p-Smad3 levels in RAW264.7 cells treated with IL-4, thereby indicating the induction of fibrosis. However, following icariin treatment, a significant reduction in the expression levels of TGF-β1, p-Smad2, and p-Smad3 was observed. This observation indicates that icariin effectively suppresses cardiac fibrosis by inhibiting the TGF‐β1, p-Smad2, and p-Smad3 signaling pathways in M2-type macrophages (see Fig. 6).

## Discussion

The findings presented in this study provide compelling evidence that the administration of icariin effectively mitigates cardiomyocyte fibrosis and leads to a significant improvement in cardiac function. Through its regulation of the TGF‐β/Smad signaling pathway, icariin exhibits the capacity to enhance ventricular reconstruction and inhibit myocardial fibrosis in rats with MI. Furthermore, the study indicates that icariin holds promise as a potential therapeutic target for addressing cardiac fibrosis.

In recent times, emergency thrombolysis and interventional therapy have witnessed a surge in popularity and widespread implementation. These advancements have not only improved the prognosis of patients with myocardial infarction but have also positively impacted their quality of life [21]. Although most patients achieve complete recovery, a subset may develop distressing symptoms such as chest pain, respiratory difficulties, arrhythmias, or even heart failure, necessitating hospitalization. The development and progression of ventricular dysfunction [22, 23], arrhythmias [24, 25], and unfavorable outcomes in heart failure (HF) patients are closely associated with ventricular remodeling [26]. In this regard, developing novel treatments is a problem that needs to be addressed urgently. In recent years, the relationship between TCM and cardiovascular disease has received increased attention [27–29]. TCM compounds are known to comprise a diverse array of Chinese medicinal ingredients that act through various targets, channels, and pathways to address diseases. Among these, icariin, the primary active ingredient derived from the Chinese herbal medicine Epimedium brevicornu Maxim, has garnered considerable interest [30, 31]. Several species of Epimedium can be used to extract it, including Epimedium sagitta, Epimedium pilose, Epimedium Wushan, and Epimedium korean. Icariin has been shown to protect the myocardium from ischemia and reperfusion in previous studies [32–35]. Icariin has a therapeutic effect on coronary heart disease, but its mechanism remains largely unclear. According to TCM principles, ventricular dysfunction and heart failure arise from Qi deficiency and blood stasis [36, 37]. In this study, icariin therapy exhibited notable improvements in LVEF and FS while concurrently reducing LVEDD and LVESD compared to the MI group. Moreover, this study sheds light on the hitherto unexplored function and mechanism of icariin in mitigating cardiac fibrosis.

The TGF-β1/Smad signaling pathway assumes a pivotal role in cardiac fibrosis and ventricular remodeling [38]. This study sought to quantify the changes in the expression levels of key molecules within the TGF-β1 signaling pathway. Notably, following MI, there was evident overexpression of TGF-β1 in the myocardium, whereas treatment with icariin resulted in a significant reduction in TGF-β1 in the ischemic myocardium. To validate this hypothesis, Western blotting was performed on M2-type macrophages induced by IL4, revealing that icariin supplementation led to decreased levels of TGF-β1 and Smad2/3. Presently, research concerning the antitumor activity of icariin has emerged as a prominent area of interest. These findings demonstrate that icariin effectively inhibits M2 macrophage polarization, signifying its potential to modulate the tumor microenvironment. Prior studies have already established icariin's ability to inhibit M2 macrophage polarization while promoting M1 macrophage polarization [18, 39]. There is still work to be done to determine whether icariin affects myocardial fibrosis and ventricular remodeling by regulating macrophage polarization.

In conclusion, the present study provides compelling evidence that icariin significantly reduces cardiac fibrosis and ameliorates cardiac function in rats with myocardial infarction. The potential protective effects of icariin are closely associated with modulation of the TGF-β1/Smad signaling pathway. However, it is essential to acknowledge certain limitations within this study. First, the follow-up period for the MI rats was relatively short, thereby precluding the observation of long-term changes in cardiac function. Second, given the intricate molecular mechanisms involved, the precise mechanisms through which icariin confers protective effects on cardiac fibrosis remain incompletely elucidated. Thus, further investigations are warranted to validate and expand upon the present findings.

## Limitations of the study

Firstly, the selection of icariin dosage in our study was based on a review of pertinent literature. However, it is noteworthy that there is a dearth of dose–response investigations aimed at determining the most efficacious icariin dosage for achieving optimal outcomes. Additionally, comprehensive long-term safety evaluations for icariin treatment are presently lacking. Secondly, we did not employ TTC staining to validate the myocardial infarction's extent. Lastly, it should be acknowledged that this study was characterized by a limited sample size, an absence of power calculations, and the potential influence of uncontrolled confounding variables.

## Conclusion

The findings of the current study suggest that icariin possesses the capacity to enhance cardiac function and mitigate ventricular remodeling in rats following MI. These results underscore icariin's potential as a viable therapeutic target for addressing myocardial injury and ventricular remodeling.

## Data Availability

All data and materials utilized in this study are accessible upon reasonable request from the corresponding author.

## Abbreviations

MI: Myocardial infarction
LAD: Left anterior descending coronary artery
LVEDD: Left ventricular end-diastolic diameter
LVESD: Left ventricular end-systolic diameter
FS: Left ventricular shortening fraction
EF: Left ventricular ejection fraction
ACEI: Angiotensin-converting enzyme inhibitors
ARB: Angiotensin receptor blockers
RAAS: Renin–angiotensin–aldosterone system
MMPs: Matrix metalloproteinases
TCM: Traditional Chinese medicine
ELISA: Enzyme-linked immunosorbent assay
SD: Sprague–Dawley
H&E: Hematoxylin and eosin
DMSO: Dimethyl sulfoxide;
ANOVA: One-way analysis of variance
TGF-β: Transforming growth factor beta
ECM: Matrix metalloproteinases
p‐Smad2: Phosphorylated Smad2
p‐Smad2: Phosphorylated Smad3
LV: Left ventricular
